# Rapid Increase in Prevalence of Male Circumcision in Rural Tanzania in the Absence of a Promotional Campaign

**DOI:** 10.1371/journal.pone.0040507

**Published:** 2012-07-06

**Authors:** Harriet J. Forbes, Aoife M. Doyle, Kaballa Maganja, John Changalucha, Helen A. Weiss, David A. Ross, Richard J. Hayes

**Affiliations:** 1 London School of Hygiene and Tropical Medicine, London, United Kingdom; 2 National Institute for Medical Research, Mwanza Centre, Mwanza, Tanzania; Yale School of Public Health, United States of America

## Abstract

**Objectives:**

To estimate the prevalence of circumcision among young men in rural Mwanza, North-Western Tanzania, and document trends in circumcision prevalence over time. To investigate associations of circumcision with socio-demographic characteristics, reported sexual behaviours and sexually transmitted infections (STIs).

**Design:**

A cross-sectional survey in communities which had previously participated in a cluster-randomized trial of an adolescent sexual health intervention that did not include male circumcision in 20 rural communities.

**Methods:**

In 2007/08, 7300 young men (age 16–23 years) were interviewed and examined by a clinician. The prevalence of circumcision by age was compared with data collected during the trial in 1998–2002. Odds ratios (OR) and 95% confidence intervals (CI) for the association of circumcision with socio-demographic characteristics, reported sexual behaviours and with HIV and other STIs were estimated using multivariable conditional logistic regression.

**Results:**

The prevalence of male circumcision was 40.6%, and age-specific prevalence had more than doubled since 2001/2002. Circumcised men reported less risky sexual behaviours, being more likely to report having ever used a condom (adjusted OR = 2.62, 95%CI:2.32–2.95). Men circumcised before sexual debut were at reduced risk of being HIV seropositive compared with non-circumcised men (adjusted OR = 0.50, 95%CI:0.25–0.97), and also had reduced risks of HSV-2 infection and genital ulcer syndrome in the past 12 months compared with non-circumcised men.

**Conclusions:**

There was a steep increase in circumcision prevalence between 2001/02 and 2007/08 in the absence of a promotional campaign. Circumcised men reported safer sexual practices than non-circumcised men and had lower prevalence of HIV and HSV-2 infection.

## Introduction

Evidence from three randomized controlled trials (RCTs) has established that male circumcision reduces the risk of acquisition of HIV infection through heterosexual intercourse by 50–60% [1], [2], [3]. There is also some evidence that circumcision protects against other sexually transmitted infections (STIs), particularly ulcerative STIs (Herpes simplex virus type-2 (HSV-2) [4], [5], [6], [7] and chancroid [4]). STIs are cofactors that enhance both the acquisition and transmission of HIV [8], [9], and circumcision may act to reduce HIV acquisition indirectly as well as directly.

In 2007 the World Health Organization (WHO) and the Joint United Nations Programme on HIV/AIDS (UNAIDS) declared that circumcision should be considered an important additional intervention for HIV prevention [10]. The current focus is on scaling-up circumcision services in areas where HIV prevalence is high and male circumcision prevalence is low. This requires an understanding of current and recent trends in circumcision prevalence.

In Tanzania, prevalence of male circumcision varies substantially by region, with the lowest prevalence in North Western and Western Tanzania (around 24%) and highest in eastern Tanzania (over 95%) [11]. Our study took place in Mwanza Region, North-Western Tanzania, where circumcision prevalence was estimated at 56% in 2007/08 [11]. The main ethnic group in North-Western Tanzania, the Sukuma, are traditionally non-circumcising, though studies from the 1990s suggested that circumcision was increasing among them [12], [13]. It is typically performed in the late teens and early 20s [12]. In addition to ethnicity and religion, factors associated with circumcision in North-Western Tanzania are higher levels of education and urban location [13].

In this paper, we analyze data from a cluster-randomized trial of an adolescent sexual health intervention [14] to investigate patterns of male circumcision among young men living in rural areas of Mwanza Region. Our objectives were to report the prevalence and determinants of circumcision, its association with reported sexual behaviours and laboratory-identified STIs and changes in its prevalence over time, in order to provide guidance to current and future circumcision promotion initiatives in this Region.

## Methods

### Study Design

Data for this study came from the MEMA kwa Vijana Trial Further Survey (MkV1FS), a cross-sectional survey carried out in 2007/08 among young people from 20 rural communities in Mwanza Region, Tanzania. The 2007/08 survey was designed to assess the long-term impact of a package of adolescent sexual and reproductive health interventions aiming to reduce the incidence of HIV, STIs and unintended pregnancies, within a cluster-randomized trial [15] in which 10 intervention communities were compared with 10 comparison communities. Trial interventions did not discuss or promote male circumcision [16]. During the cluster-randomized trial, data were collected on a cohort of 9,645 young people at baseline (in 1998), and at approximately 18 and 36 months after the start of the interventions, in 2000 and 2001/02 respectively. Data from these three trial surveys were compared with the 2007/8 survey to analyse trends in circumcision prevalence from 1998–2007. Full details of the cluster-randomized trial design and results have been published previously [15], [16], [17].

### Study Population

Between July 2007 and May 2008, eligible young people for MkV1FS were identified during a household census in each of the 20 trial communities, and were invited to participate. Eligible participants had attended at least one of school years 5–7 within one of the trial communities between 1999–2002 (when the intervention was implemented most intensively). In order to capture more eligible participants, the communities, nearby schools and major migration points within the Lake Zone of Tanzania were revisited in June-July 2008.

### Survey Methods

Consenting participants for MkV1FS were interviewed at a central location in their village using standardised face-to-face questionnaires to collect information on lifestyle, health and socio-demographic factors. Circumcision status was ascertained by self-report and a physical examination by a study clinician. Blood and urine samples were collected to test for HIV, HSV-2, chlamydia, gonorrhoea and syphilis. If positive for lifetime syphilis [defined as Serodia *Treponema pallidum* particle agglutination (TPPA) test positive] they were further tested for active syphilis using the Immutrep carbon antigen rapid plasma reagin (RPR) test [14]. Participants were asked if they had experienced genital ulcer syndrome (GUS) or symptoms of abnormal genital discharge during the past 12 months.

### Statistical Analysis

Data were analyzed using STATA 11.0. Unless specified otherwise, circumcision was defined using clinician-diagnosis rather than self-report.

Age-specific prevalence of circumcision was analyzed among men seen at the MkV1FS and compared with prevalence at the three previous surveys carried out during the trial. The age-specific prevalence of self-reported circumcision was compared between the 1998 survey, the 2000 survey and the MkV1FS survey (self-reported circumcision status was not asked at the 2001/02 survey), to substantiate any trends found in clinician-diagnosed circumcision prevalence. To examine whether there had been a change in age at circumcision, self-reported age at circumcision was analyzed for men currently aged 20, 21–22, 23–24 and 25+ years at the MkV1FS, restricting analysis to those circumcised at/before 20 years of age. Circumcision status for participants interviewed at both the final trial survey (2001/02) and the MkV1FS (2007/08) was analyzed to assess the number circumcised between the two surveys.

 Conditional logistic regression was used to estimate odds ratios (OR) and 95% confidence intervals (CI) for associations between circumcision status and socio-demographic factors in the MkV1FS, using the clogit command. This analysis was conditioned on study community because this adjusts fully for any confounding effects of community, and allows for clustering by community, without the need for any assumptions about the distributional form of the between-community variation. A multivariable risk-factor model was built as follows: variables were added, starting with those most strongly related to circumcision in univariable analyses until no further variables significantly improved the model’s fit, assessed with the likelihood ratio test (P<0.10). For collinear variables, the variable considered a-priori to be most likely to be a risk factor (from previous research) was kept in the model.

Since effects of male circumcision on HIV and other STIs may depend on whether men were circumcised before or after sexual debut, associations with biological outcomes were examined with circumcision status in three categories: ‘non-circumcised’, ‘circumcised at/after sexual debut’ or ‘circumcised before sexual debut’, based on self-reported age at circumcision and age at sexual debut. To analyse the effects of male circumcision status on sexual behaviour, circumcision was kept as a binary variable. Conditional logistic regression was used to estimate the OR for the association between each STI outcome or sexual behaviour outcome, and circumcision status in the MkV1FS. Since the objective of this analysis was to examine the effects of a single exposure (circumcision status) rather than to build a general risk factor model, these analyses were adjusted only for age (considered an *a-priori* potential confounder, because younger men were more likely to be circumcised and less likely to have HIV) and any other variables identified as confounders (if their inclusion changed the age-adjusted OR for the association between circumcision and the outcome by 10% or more). Sensitivity analyses were conducted to investigate the potential effects of missing data on the association of circumcision status and biological outcomes.

### Ethical Considerations

The MkV trial and further survey were approved by the LSHTM Ethics Committee and the Medical Research Coordinating Committee in Tanzania. For the MkV trial and further survey signed informed consent was obtained from each participant on the day of the survey round. In the further survey, additional written consent from parents was obtained for participants under the age of 18 years.

## Results

In total 7,300 males were eligible and enrolled in the in the MkV1FS. Full details on the number of individuals attending the census and survey have been published previously [14].

### Characteristics of the Study Population

The male participants were aged 15–34 years (median age 22 years). Most (78.4%) belonged to the Sukuma ethnic group and most were Christian (80.7%) (Table 1). Farming was the most common occupation (45.8%), followed by being at school or university (23.9%). Most participants (91.0%) were sexually active (Table 2). Among sexually active males, the median number of lifetime partners reported was 4 (Inter quartile range (IQR): 2–4), median reported age at sexual debut was 17 years (IQR: 15–18 years) and 63.4% reported ever having used a condom. HIV prevalence was 1.8%, HSV-2 prevalence was 25.8%, 3.5% tested positive for active syphilis, and 6.0% reported GUS during the past 12 months. No substantial socio-demographic differences were identified between the 7,177 participants who did and 123 who did not have their circumcision status assessed by a study clinician [data not shown].

**Table 1:** Socio-demographic characteristics among 7300 male participants in the MkV further survey, and their associations with male circumcision.

Variable	Number ofmen (%)	Numbercircumcised (%)	UnadjOR (95%CI)	Adjusted* OR
All Participants	7300 (100)	2911 (40.6^1^)	–	–
Group^2^				
Comparison	3494 (47.9)	1316 (38.3)	–	–
Intervention	3806 (52.1)	1595 (42.6)	–	–
Age				
<21	2046 (28.0)	915 (45.6)	1 P-trend<0.01	1 P-trend<0.01
21–22	1977 (27.0)	787 (40.7)	0.77 (0.67–0.88)	0.80 (0.69–0.93)
23–24	1914 (26.2)	730 (38.6)	0.68 (0.59–0.78)	0.74 (0.64–0.85)
25+	1362 (18.7)	478 (35.6)	0.54 (0.47–0.64)	0.63 (0.53–0.74)
Ethnic Group				
Non-Sukuma	1575 (21.6)	975 (63.2)	1 P<0.01	1 P<0.01
Sukuma	5716 (78.4)	1934 (34.4)	0.39 (0.33–0.45)	0.46 (0.40–0.54)
Religion				
Christian	5883 (80.7)	2506 (43.3)	1 P<0.01	1 P<0.01
Moslem	330 (4.5)	262 (80.6)	6.97 (5.19–9.35)	6.06 (4.50–8.16)
Other Religion/No religion	1076 (14.8)	139 (13.1)	0.26 (0.21–0.32)	0.29 (0.24–0.35)
Highest level of education reached^3^				
Primary or less	5096 (69.9)	1590 (31.8)	1 P<0.01	1 P<0.01
Secondary or higher	2196 (30.1)	1319 (61.0)	3.57 (3.18–4.01)	3.12 (2.74–3.55)
Occupation				
Farmer	3333 (45.8)	884 (27.0)	1 P<0.01	1 P<0.01
At School/University	1740 (23.9)	1070 (62.6)	4.37 (3.81–5.00)	3.67 (3.15–4.28)
Petty Trade	1047 (14.4)	318 (31.0)	1.25 (1.05–1.48)	1.12 (0.94–1.33)
Fisherman	300 (4.1)	212 (70.7)	2.04 (1.52–2.74)	1.73 (1.27–2.35)
Mine Employee	233 (3.2)	89 (39.0)	1.80 (1.33–2.43)	1.51 (1.11–2.07)
Other	623 (8.6)	326 (53.1)	3.10 (2.56–3.75)	2.48 (2.04–3.03)
Marital Status^4^				
Married	2444 (33.5)	689 (28.6)	1 P<0.01	1 P<0.01
Separated/Widowed/Divorced	229 (3.1)	77 (33.9)	1.32 (0.96–1.82)	1.20 (0.86–1.69)
Never Married	4627 (63.4)	2145 (47.2)	2.38 (2.12–2.68)	2.13 (1.85–2.45)

* Adjusted for age, ethnic group and religion.

^1^Of 7300 surveyed, 123 males had missing data for circumcision status. ^2^ Odds ratios not calculable for intervention and comparison group as model is conditional on community. ^3^8 missing values ^4^34 missing values.

**Table 2:** Association between male circumcision status and reported sexual behaviour in 7177 male participants in the MkV further survey.

Variable	Category	Prevalence % (No/total)	UnadjOR (95%CI)	Age-adjOR (95%CI)
***All participants (N = 7177)***				
**Sexually active**	Non-circumcised	91.4 (3894/4261)	1 P = 0.16	1 P = 0.46
	Circumcised	90.6 (2635/2909)	0.88 (0.74–1.05)	1.07 (0.89–1.29)
**≥5 lifetime sexual partners**	Non-circumcised	42.0 (1781/4245)	1 P<0.01	1 P = 0.46
	Circumcised	38.5 (1115/2895)	0.84 (0.76–0.94)	0.96 (0.86–1.07)
***Sexually Active Participants (N = 6529)***				
**Age at sexual debut** **<16 (years)**	Non-circumcised	30.5 (1183/3878)	1 P = 0.03	1 P<0.01
	Circumcised	26.6 (698/2622)	0.88 (0.78–0.99)	0.83 (0.74–0.94)
**Ever used a condom**	Non-circumcised	55.0 (2141/3890)	1 P<0.01	1 P<0.01
	Circumcised	75.9 (2000/2635)	2.52 (2.23–2.84)	2.62 (2.32–2.95)
**≥3 sexual partners in** **last 12 months**	Non-circumcised	27.0 (1047/3880)	1 P = 0.24	1 P = 0.60
	Circumcised	24.8 (652/2631)	0.93 (0.82–1.05)	0.97 (0.85–1.10)
***Participants sexually active in last 12 months (*** **N = 5763)**				
**>1 partner in last 4 weeks**	Non-circumcised	16.5 (569/3446)	1 P = 0.28	1 P = 0.53
	Circumcised	14.1 (317/2252)	0.91 (0.78–1.08)	0.95 (0.80–1.12)
**Used condom with last** **sexual partner**	Non-circumcised	23.1 (790/3428)	1 P<0.01	1 P<0.01
	Circumcised	44.6 (1002/2245)	2.88 (2.54–3.28)	2.76 (2.42–3.14)
***Participants with non-regular partner in last 12 months (*** **N = 3509)**				
**Used condom with last** **non-regular partner**	Non-circumcised	37.8 (783/2073)	1 P<0.01	1 P<0.01
	Circumcised	59.8 (859/1436)	2.63 (2.25–3.07)	2.64 (2.26–3.09)

Note: Missing values for reported sexual behaviours ranged from 4 to123.

### Prevalence and Incidence of Male Circumcision by Age

**Figure 1:**
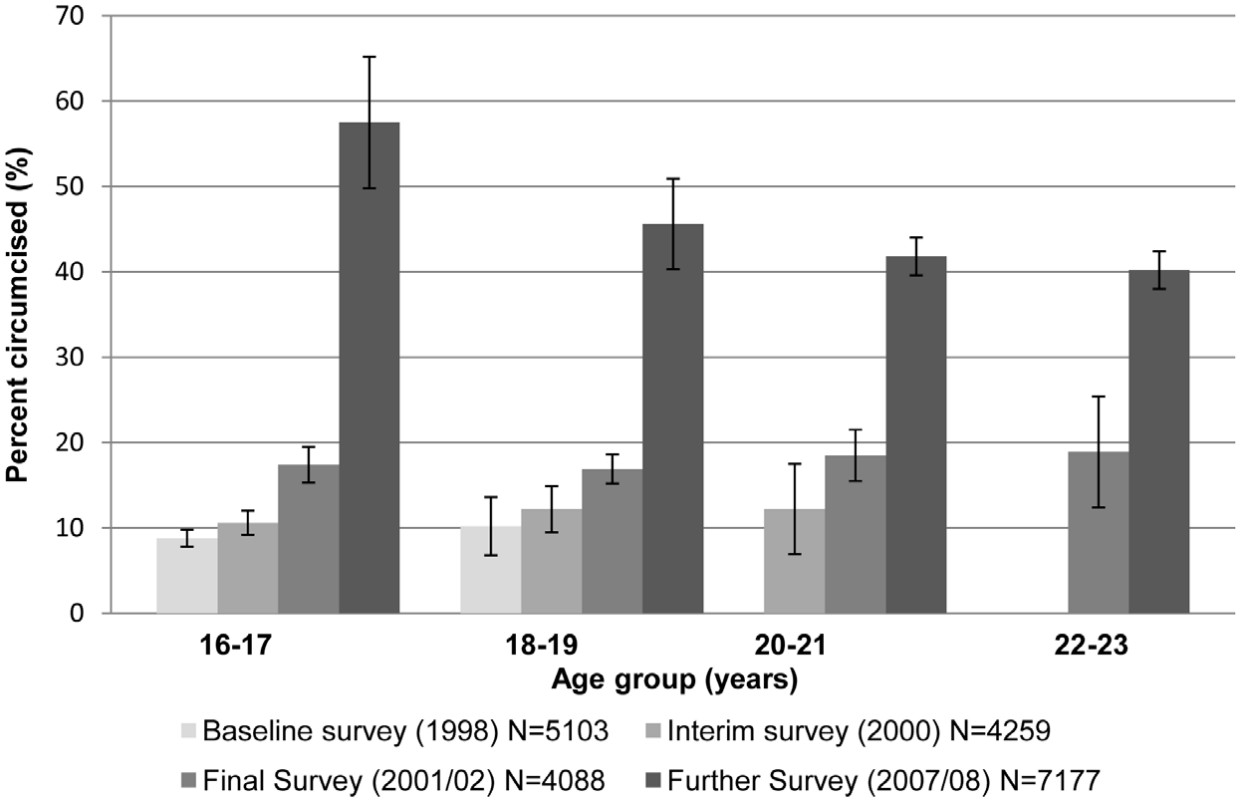
Prevalence of circumcision according to current age at each of the four surveys (error bars show 95% CIs).

Overall 2,911 males (40.6%) were judged by the study clinicians to have been circumcised. Younger age was strongly associated with circumcision, with prevalence of circumcision decreasing from 45.6% among those aged under 21 years to 35.6% of those 25 years or more (p-trend<0.0001) (Table 1), suggesting increased prevalence of circumcision over time. This is supported by a comparison of prevalence across the three surveys carried out during the cluster-randomized trial from 1998–2002 and in the MkV1FS in 2007/8(Figure 1). Between the 1998 and 2001/02 surveys, there was a small increase in prevalence but by 2007/08, the prevalence had more than doubled among men at all ages (Figure 1). This is supported by self-reported circumcision prevalence; among men aged 18 years in each of the survey rounds, 13.7% self-reported they were circumcised in the 1998 survey, 18.4% in the 2000 survey and 52.6% at the 2007/08 survey.

Overall 1,974 men were seen at both the 2001/02 and 2007/08 surveys, and had circumcision status recorded at both surveys. Among these, 749 were circumcised at the time of the 2007/08 survey and, 472 (63%) had been circumcised since 2002 [data not shown]. This indicates a large number of young men were circumcised between 2001/02 and 2007/08.

**Figure 2:**
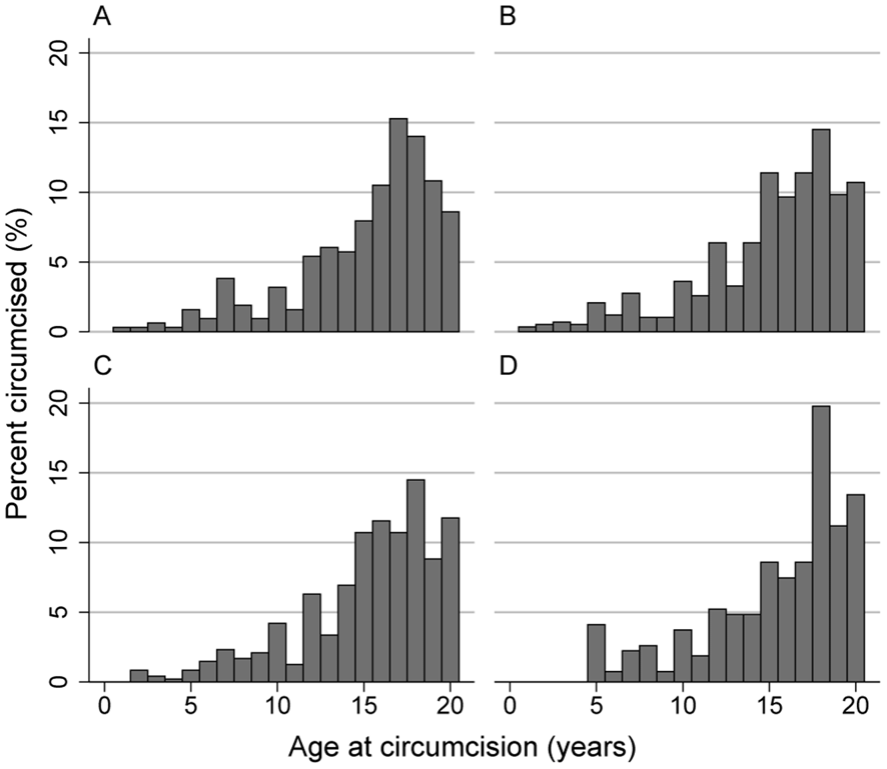
Distribution of age at circumcision by current age. Current age is age in 2007/08 survey and sample is restricted to those circumcised at/before 20 years of age and who were at least 20 years old at the further survey. A) <21 years (N = 314) B) 21–22 years (N = 579) C) 23–24 years (N = 476) D) 25+ years (N = 268). *Median is median age at circumcision within the current-age birth cohort, IQR is inter-quartile range.

At the MkV1FS, reported age at circumcision was available for 2,338 men (80.3%), with median age at circumcision 16 years (IQR13–19). There was some indication that age at circumcision was younger among those aged less than 21 years compared with those aged 25 years and over (Figure 2).

### Socio-demographic Factors Associated with Male Circumcision

Apart from the strong association with age, several other socio-demographic factors showed an association with circumcision in the MkV1FS (Table 1). Sukuma men were less likely to be circumcised than non-Sukuma men (34.4% vs 63.2%; adjusted OR (adjOR) = 0.46, 95%CI:0.40–0.54). As expected, the majority of Muslims were circumcised (80.7%), compared with 43.3% of Christians (adjOR = 6.06, 95%CI:4.50–8.16). The prevalence of circumcision was lowest among males with ‘No religion/Other religion’ (13.1%). Farmers had the lowest prevalence of circumcision (27.0%) whilst fishermen (70.7%) and those at school/university (62.6%) had the highest. Circumcision was more prevalent among never married men than married men (adjOR = 2.13, 95%CI 1.85–2.45), and among those with secondary education (adjOR = 3.12, 95%CI: 2.74–3.55). There was little difference in circumcision prevalence between the intervention and comparison communities (42.6% vs 38.3%). Occupation, marital status and educational group were not included in the final model because of their collinearity with age.

### Association of Male Circumcision with Sexual Behaviours

Table 2 shows some evidence that being circumcised was associated with lower-risk reported behaviours among sexually active men in the MkV1FS. Circumcised men were less likely to report an early age at sexual debut (<16 years) (26.6% vs 30.5%; adjOR = 0.83, 95%CI:0.74–0.95), and more likely to report ever having used a condom (75.9% vs 55.0%; adjOR = 2.62, 95%CI:2.32–2.95). There was no evidence of a difference in number of reported lifetime or recent partners by circumcision status (Table 2). None of the variables in Table 1 confounded the association between circumcision and sexual behaviours; multivariable analyses were therefore adjusted only for age.

### Association of Male Circumcision with HIV and Other STIs

These analyses were restricted to 6,672 (93%) of the 7,177 participants with clinician-assessed circumcision status in the MkV1FS, for whom both the reported age at sexual debut and age at circumcision were known (Table 3). HIV prevalence was lowest among men circumcised before sexual debut (0.9% vs 2.0% in non-circumcised men; adjOR = 0.50, 95%CI 0.25–0.97) while there was little evidence of a protective effect among men circumcised after sexual debut (adjOR = 0.85, 95%CI 0.52–1.40). There was evidence of a protective effect of circumcision on HSV-2 among men circumcised before sexual debut (adjOR = 0.67, 95%CI:0.57–0.80), and a weaker effect among those circumcised after sexual debut (adjOR = 0.78, 95%CI:0.66–0.92). Unadjusted analyses showed evidence that being circumcised before sexual debut was associated with lower odds of having lifetime or active syphilis, compared to being non-circumcised, but this did not persist after adjusting for confounders (Table 3). Men circumcised before sexual debut were also at lower risk of reporting genital ulcer syndrome (GUS) in the last 12 months compared with non-circumcised men (adjOR = 0.69, 95%CI:0.47–1.00). There was little evidence of an association of circumcision status with chlamydial or gonorrhoeal infections, or with symptoms of abnormal genital discharge in the past 12 months.

**Table 3:** Male circumcision status and risk of HIV and other STIs among 6672 male participants in the MkV further survey.

Outcome		Prevalence % (No/total)		UnadjOR (95%CI)		AdjOR (95%CI)
**HIV seropositive**						
Non-circumcised		2.0 (85/4248)		1 P = 0.01		1 P = 0.09^1^
Circumcised at/after sexual debut		2.2 (23/1050)		0.94 (0.57–1.53)		0.85 (0.52–1.40)
Circumcised before sexual debut		0.9 (12/1347)		0.42 (0.22–0.80)		0.50 (0.25–0.97)
**HSV-2 seropositive**						
Non-circumcised		28.4 (1206/4248)		1 P<0.01		1 P<0.01^2^
Circumcised at/after sexual debut		24.7 (259/1050)		0.77 (0.66–0.91)		0.78 (0.66–0.92)
Circumcised before sexual debut		19.0 (256/1347)		0.58 (0.49–0.68)		0.67 (0.57–0.80)
**Lifetime syphilis [TPPA+]**						
Non-circumcised		5.7 (244/4248)		1 P = 0.05		1 P = 0.72^3^
Circumcised at/after sexual debut		6.0 (63/1050)		0.98 (0.73–1.32)		1.10 (0.81–1.50)
Circumcised before sexual debut		4.3 (58/1347)		0.69 (0.50–0.95)		0.95 (0.68–1.33)
**Active syphilis [RPR+/TPPA+]**						
Non-circumcised		3.8 (162/4248)		1 P = 0.09		1 P = 0.98^3^
Circumcised at/after sexual debut		3.5 (37/1050)		0.85 (0.58–1.24)		0.98 (0.67–1.45)
Circumcised before sexual debut		2.8 (38/1347)		0.65 (0.44–0.97)		0.96 (0.63–1.45)
**Chlamydia**						
Non-circumcised		2.3 (97/4262)		1 P = 0.48		1 P = 0.38^1^
Circumcised at/after sexual debut		2.0 (21/1054)		0.87 (0.53–1.43)		0.74 (0.44–1.22)
Circumcised before sexual debut		1.8 (24/1352)		0.74 (0.45–1.22)		0.77 (0.46–1.29)
**Gonorrhoea**						
Non-circumcised		0.4 (17/4262)		1 P = 0.77		1 P = 0.95^3^
Circumcised at/after sexual debut		0.4 (4/1054)		0.95 (0.30–3.01)		1.13 (0.34–3.72)
Circumcised before sexual debut		0.3 (4/1352)		0.65 (0.20–2.17)		0.90 (0.26–3.14)
**Symptoms of abnormal genital discharge in past 12 months**						
Non-circumcised		8.8 (375/4261)		1 P<0.01		1 P = 0.42^4^
Circumcised at/after sexual debut		9.3 (98/1053)		1.06 (0.83–1.35)		1.04 (0.80–1.35)
Circumcised before sexual debut		6.4 (86/1352)		0.67 (0.52–0.88)		0.84 (0.62–1.13)
**Symptoms of genital ulcers in past 12 months**						
Non-circumcised		6.3 (269/4259)		1 P<0.01		1 P = 0.06^4^
Circumcised at/after sexual debut		7.1 (75/1052)		1.03 (0.78–1.36)		1.08 (0.81–1.46)
Circumcised before sexual debut		3.9 (53/1352)		0.54 (0.39–0.75)		0.69 (0.47–1.00)

^1^Adjusted for age and ever used a condom, ^2^ Adjusted for age only, ^3^Adjusted for age and religion, ^4^Adjusted for age and used condom with last sexual partner.

TPPA+  =  Serodia *Treponema pallidum* particle agglutination test. RPR  =  Immutrep carbon antigen rapid plasma reagin test.

Missing values for STIs ranged from 10 to 41.

The 505 (7%) circumcised participants not included in this analysis of circumcision and STIs, because data on their age at circumcision and/or age at sexual debut were unavailable, tended to be younger and were more likely to be Muslim. In a sensitivity analysis in which the 505 participants were classified as having been circumcised before sexual debut, the protective effect of being circumcised before sexual debut on HIV was less strong (adjOR = 0.73 95%CI:0.44–1.21). Inclusion of these participants had little effect on the association of circumcision and other STIs.

## Discussion

This study suggests there was a dramatic increase in the prevalence of circumcision among young men in rural areas of Mwanza Region, Northern Tanzania, between the late 1990s and 2008 despite no active health promotion of circumcision occurring in the Region during that time period. The proportion of young men who were circumcised was 41% in 2007/08, and had more than doubled since the 2001/02 survey at all ages studied (16–23 years). The median reported age at circumcision was 16 years, with some indication of an earlier age at circumcision among the youngest birth cohorts.

The increase in circumcision prevalence is notable, particularly considering that most participants belong to the Sukuma ethnic group, who are traditionally non-circumcising, and that this occurred before active promotion of circumcision in the Region. Four independent studies carried out in rural Mwanza Region during the 1990s suggested there was a low prevalence of circumcision among adult men of 10–15% [12], [13]. Data from the 2001/02 survey within the MEMA kwa Vijana trial in rural Mwanza Region also suggested the prevalence was around 17% among men aged 16–20 [18]. Another survey in selected villages in rural Mwanza Region conducted in 2004 reported a prevalence of 30% among 15–44 year olds [19], [20]. National surveys have reported a relatively high prevalence of circumcision in Mwanza Region of around 55% in 2003 and again in 2007; however these estimates included urban areas and were based on very small sample sizes of around 300 men [11], [21]. The strength of this study in determining the prevalence of circumcision over time is that it uses data from the long-term follow-up of the same study populations, rather than comparing estimates from studies in different populations at different time periods.

This rise in prevalence in male circumcision in rural Mwanza Region is also supported by qualitative evidence from the 1990s [12], [13] indicating that circumcision was becoming more acceptable and widely practised within Mwanza Region. It was suggested that interaction with circumcising ethnic groups and changing local perceptions that associate male circumcision with modernity and sexual hygiene were driving the change in attitudes [12]. Together, the qualitative and quantitative evidence suggests that the increase in circumcision prevalence observed here among youth in rural Mwanza Region is a continuation of a trend that began in the 1990s, and has then accelerated substantially.

Since the results of the circumcision trials have been published, the Tanzanian government has developed a national circumcision strategic plan which aims to provide free circumcision to 2.8 million men and boys aged 10–34 years from 2010–2015 [22], [23]. Prior to 2008 however there were no large-scale formal circumcision promotion campaigns in Tanzania, so public health initiatives cannot have been responsible for the observed trends. The first trial evidence confirming that circumcision conferred protection against HIV was not published until 2005. It seems unlikely that this knowledge would have reached sufficient numbers of young men within this rural area by 2007/08 to have driven this increase in the absence of a publicity campaign and active promotion of circumcision. It may be that the younger generation are more health-aware and this may have led to an increase in circumcision. Qualitative work in Mwanza Region and elsewhere in East Africa shows that circumcision is perceived as a hygienic practice [12], [18], [24]. This is supported by our finding that circumcision was associated with safer sexual behaviours in this population. The rapid expansion of secondary education in most of rural Tanzania which occurred after 2001/2 may also have contributed to the observed increase in circumcision prevalence, given that higher than primary education has been associated with greater circumcision [24].

However, it is important to explore whether the observed increase in circumcision prevalence could be due to an artefact of the data, rather than a true effect. Firstly, if participants at the various surveys were not comparable, this might explain the apparent increase. However, analysis of the socio-demographic characteristics of participants in the 2001/02 and 2007/08 surveys showed no such differences [data not shown]. There were more Christians in the 2007/08 survey compared to the 2001/02 survey (80.7% versus 70.4%); self-identification as a Christian may increase with age. However, as self-identifying as a Christian did not determine circumcision status, and as there was no difference in the proportion of Muslims, this is not likely to have driven the increase.

Secondly, if study clinicians were better at recognising circumcised men at the 2007/08 survey, this could have led to an apparent increase in circumcision prevalence. Problems of clinician-assessed circumcision status have been raised, particularly regarding the difficulty in recognising partial circumcisions [18]. However, the training given to the clinicians was similar for each of the MEMA kwa Vijana surveys. A comparison of self-reported and clinician-assessed circumcision showed a reasonably high level of agreement in the 1998 survey (97.1% concurrence) [18]. Moreover, similar trends were obtained when analysing self-reported circumcision status. It therefore seems unlikely that the observed increase in male circumcision is attributable to differential reporting of circumcision by clinicians.

Within our study cohort, circumcised men reported less risky sexual behaviour, particularly regarding condom use. In contrast, a cross-sectional study in Mbale, Uganda, found circumcised men engaged in riskier sexual behaviours; circumcised men had more extra-marital partners, and more sex in exchange for gifts or money [25]. However a cohort study in Western Kenya found no evidence for any difference in risky behaviours between recently circumcised and non-circumcised men [26], and a review of studies from Mwanza Region found condom use tended to be higher in circumcised men [13]. Further studies are needed to understand the sexual behaviour of circumcised compared to non-circumcised men in different settings, as behaviours associated with circumcision may be locally specific and not generalisable to other settings.

The associations between circumcision and STIs in this study are in line with results from other studies in Africa. Those circumcised before sexual debut had a 50% lower odds of having HIV, compared to non-circumcised men, comparable with the risk reduction of 50–60% found in circumcision trials [1], [2], [3]. As might be expected, there was less association with HIV among those circumcised at or after sexual debut, as it is possible that these men became infected before circumcision. Being circumcised was associated with reduced odds of having HSV-2, supporting evidence from two of the RCTs [6], [7] and previous observational studies [4], that circumcision protects against the acquisition of HSV-2. Syphilis and circumcision were not associated in this study after adjustment for confounders, which although contradicting other observational data, [4] was also seen in trial data from Uganda [3]. Among those circumcised before sexual debut, there was some evidence of an association with GUS after adjusting for confounders, which is consistent with other evidence suggesting circumcision protects against genital ulcer disease [27]. As with trial data [28], [29], there was no evidence that circumcision protects against the non-ulcerative STIs, chlamydia and gonorrhoea.

Our study had some limitations. First, the estimated age at circumcision was likely to be an approximation for many participants, limiting interpretation of age at circumcision. Missing circumcision status (with circumcision divided into three categories), for the 505 circumcised individuals in whom age at circumcision and/or age at sexual debut was unknown, could also potentially have biased the results, and a sensitivity analyses did demonstrate a less strong association between circumcision and HIV that was no longer statistically significant. There is also the possibility of reverse causality; the cross-sectional design could not establish the sequence of circumcision and STIs, and some men may have been circumcised as a result of having an STI, but this would tend to underestimate any protective effect.

In conclusion, the dramatic increase in circumcision prevalence over a relatively short period of time in this population, in the absence of any circumcision promotion campaigns, demonstrates that traditionally non-circumcising groups are amenable to change regarding their attitude toward circumcision. In this study, circumcised men reported safer, rather than riskier sexual behaviours, which is encouraging. However, our data were collected prior to widespread knowledge from the RCTs that circumcision can reduce risk of HIV infection and behavioural counselling prior to adult circumcision remains an integral and essential component of circumcision scale-up.
